# Model for End-Stage Liver Disease and Sodium Velocity Predicts Overall Survival in Nonmetastatic Hepatocellular Carcinoma Patients

**DOI:** 10.1155/2018/5681979

**Published:** 2018-11-07

**Authors:** Justin Y. Tang, Nitin Ohri, Rafi Kabarriti, Santiago Aparo, Jennifer Chuy, Sanjay Goel, Jonathan M. Schwartz, Milan Kinkhabwala, Andreas Kaubisch, Chandan Guha

**Affiliations:** ^1^Department of Radiation Oncology, Montefiore Medical Center and Albert Einstein College of Medicine, Bronx, NY, USA; ^2^Department of Medicine, Division of Medical Oncology, Montefiore Medical Center and Albert Einstein College of Medicine, Bronx, NY, USA; ^3^Department of Gastroenterology and Liver Diseases, Montefiore Medical Center and Albert Einstein College of Medicine, Bronx, NY, USA; ^4^Department of Surgery, Montefiore Medical Center and Albert Einstein College of Medicine, Bronx, NY, USA

## Abstract

**Background & Aims:**

The significance of short-term changes in model for end-stage liver disease and Sodium (MELD-Na) following hepatocellular carcinoma (HCC) diagnosis is unknown. In this report, we explore the value of the rate of short-term changes in MELD-Na as an independent predictor of mortality in patients with nonmetastatic HCC.

**Methods:**

We reviewed a cohort of patients diagnosed with nonmetastatic HCC at our institution between 2001 and 2011. We evaluated potential predictors of overall survival, including baseline MELD-Na and the change in MELD-Na over 90 days. We explored survival times of cohorts grouped by baseline MELD-Na and the change in MELD-Na.

**Results:**

182 patients met eligibility criteria. With a median follow-up of 21 months for surviving patients, 110 deaths were observed (60%). Median MELD-Na at the time of diagnosis was 9.7 (IQR 7.5 to 13.9). The median changes in percentage of MELD-Na over 90 days were an increase of 9% (IQR -4% to 55%). Multivariable Cox proportional hazards modeling demonstrated that both baseline MELD-Na (HR=1.07 per unit increase, 95% CI 1.03 to 1.11, p<0.001) and changes in MELD-Na exceeding 40% (HR=3.69, 95% CI 2.39 to 5.69, p<0.001) were independently associated with increased mortality risk. Median survival among patients whose changes in MELD-Na were greater than 40% was 4.5 months, and median survival among the 131 other patients was 25.8 months (p<0.001).

**Conclusions:**

We identified a subset of HCC patients who have extremely poor prognosis by incorporating the rate of short-term change in MELD-Na to baseline MELD-Na score.

## 1. Introduction

In 2013, the American Cancer Society estimated that over 30,000 new cases of hepatocellular carcinoma (HCC) were diagnosed annually in the United States. The incidence of HCC in the United States is increasing [1–3]. Individuals with HCC in the United States have a 5-year survival rate of only 8.9% despite aggressive treatment [2]. Liver transplantation and local resection remain the preferred curative treatment options for early stage disease [3, 4].

The model for end-stage liver disease (MELD) is a scoring system used to predict three-month mortality in patients with advanced liver disease. It is calculated from three biochemical variables that are markers for direct and indirect measures of hepatic functions: creatinine, prothrombin time (INR), and serum bilirubin. Rising MELD scores indicate deterioration in liver function [5, 6]. The MELD (and PELD for pediatrics) scoring system was incorporated into liver transplant allocation in order to stratify patients on waiting list according to the severity of illness [7]. More recently, MELD was explored as a means of predicting prognosis in HCC patients [8, 9], building on the previous work of investigators who incorporated severity of liver disease in cancer staging (Barcelona). Furthermore, the predictive value of MELD was improved with the addition of serum sodium in the equation (MELD-Na), based on the observation that dilutional hyponatremia is a poor prognostic sign in advanced cirrhosis and portal hypertension [10, 11]. This was established in a cohort of HCC patients who were predominantly Asian and infected with hepatitis B (HBV) [11].

Our cancer center population differs from the original study population in which MELD-Na was established with respect to race, socioeconomic factors, and HCC etiologies. While HBV infection is more prevalent worldwide, hepatitis C (HCV) is more prevalent in the United States [1, 3]. Recent studies have demonstrated differences in clinical features and prognosis between HBV and HCV-induced HCC [12–14]. The applicability of MELD-Na in the setting of HCV-induced HCC warrants further evaluation. In addition, we hypothesized that the velocity of short-term changes in MELD-Na will give additional prognostic information in this setting.

The aim of this study was to analyze MELD-Na and changes in MELD-Na as prognostic markers of survival in patients with nonmetastatic HCC using a retrospective cohort from a single large US center.

## 2. Patients and Methods

### 2.1. Patient Selection

This study was approved by the Albert Einstein College of Medicine Institutional Review Board (IRB). The IRB waived the informed consent process as the study was retrospective and patient identifiers were deidentified. We utilized the Montefiore Medical Center (Bronx, NY) cancer registry to identify patients diagnosed with HCC between 2001 and 2011. Patients with a diagnosis of hepatocellular carcinoma with sufficient data to calculate MELD-Na at the time of diagnosis (within 30 days) and approximately 90 days after diagnosis (MELD-Na_90) were included in the study. Patient characteristics were tabulated using Clinical Looking Glass (CLG); an electronic medical record research tool developed at our institution [15]. Date of death was collected using social security death registry, and data was censored at the end of 2011, which is the last date that the full social security death registry is available. Patients who underwent liver transplantation were excluded, as were patients with extrahepatic (Stage IV) disease. The patient data used to support the findings of this study are restricted by the Albert Einstein College of Medicine Institutional Review Board in order to protect patient privacy. Data are available for researchers who meet the criteria for access to confidential data.

### 2.2. Measures

Abstracted data included age at diagnosis, gender, race (categorized as White, Black, Asian, and others), ethnicity (Hispanic and non-Hispanic), HCV infection (defined as either having ICD9 diagnosis, positive HCV antibody by enzyme immunoassay or detectable HCV RNA by PCR), HBV infection (defined as either having ICD9 diagnosis, positive HBV core antigen, or detectable HBV DNA by polymerase chain reaction), alcohol use (defined as ICD9 documentation of alcohol abuse or documentation of alcohol use), alpha fetoprotein (AFP) level at diagnosis (defined as AFP level drawn within 30 days of HCC diagnosis), tumor stage (defined by American Joint Committee on Cancer Criteria version 6.0), and treatment modalities (systemic treatment, hepatectomy, transplantation, TACE, or RFA). Bilirubin, creatinine, INR, and sodium values from within 30 days of HCC diagnosis and 90 days after HCC diagnosis were tabulated and used to calculate MELD-Na and MELD-Na_90, respectively.

Each MELD score was calculated using the formula: 3.78*∗*ln(bilirubin) + 11.2*∗*ln(INR) + 9.57*∗*ln(creatinine) + 6.43. The minimal value of bilirubin, INR, and creatinine was changed to 1 in the score calculation according to convention. Likewise, the maximal value of creatinine was capped at 4. MELD-Na score was calculated using the formula: MELD + 1.59*∗*(135-sodium) with serum sodium minimum and maximum range of 120-135 mEq/L [11]. The velocity of short-term changes in MELD-Na was calculated as the percent change from baseline MELD-Na scores to MELD-Na_90 scores.

### 2.3. Statistical Analyses

Descriptive statistics were used to report patient characteristics. AFP at the time of diagnosis was dichotomized at 400 ng/ml, which has been demonstrated to be an important prognostic cutoff in the past [16].

Univariate Cox proportional hazards models were built to identify associations between baseline clinical variables and overall survival. A multivariable model was built utilizing a backwards stepwise approach. Kaplan-Meier curves for overall survival were generated after dividing patients into groups of equal sizes based on baseline MELD-Na. Statistical comparisons were performed using log-rank testing.

We tested cut-points for the changes in MELD-Na increase ranging from 0% to 100% (in 5% intervals) as predictors of mortality in univariate Cox proportional hazards models. The cut-point that yielded the lowest p value, with the stipulation that at least 25% of patients fell above and below the cut-point, was selected as the optimal value. This binary variable was tested in univariate and multivariable survival models that included clinical characteristics and baseline MELD-Na. Additional variables were added to the final multivariable model to test for interactions between statistically significant independent variables as predictors of survival. Kaplan-Meier curves for overall survival were generated after dividing patients into two groups based on the optimal cutoff for MELD-Na increase. Statistical comparisons were performed using log-rank testing. Kaplan-Meier curves were also generated after dividing patients into four groups based on baseline MELD-N (below/above median) and MELD-Na increase (below/above optimal cutoff).

Statistical analyses were performed using STATA 12.1 (StataCorp, College Station, TX) and Matlab 8.4 (The Mathworks, Natick, MA). All reported p values were two-sided, and a p value cutoff of 0.05 was used to determine statistical significance. Survival times were calculated from the date of diagnosis.

## 3. Results

### 3.1. Patient Characteristics

182 HCC patients met all eligibility criteria and are included in the present analysis. Patient characteristics are summarized in Table 1. The mean age was 62, and 73% of patients were male. 54% of patients were Hispanic, and 66% had HCV infection as the sole etiology of liver disease. 66% underwent TACE and/or RFA, 39% received systemic therapy, and only 10% underwent resection. Median follow-up for all patients was 12.3 months. For surviving patients, median follow-up was 21.1 months.

### 3.2. MELD-Na and MELD-Na_90 Scores

The median baseline MELD-Na was 9.7 (IQR, 7.5-13.9), and median MELD-Na_90 was 11.8 (IQR, 8.5-18.8). MELD-Na_90 was higher than MELD-Na for 113 patients (62%), and the median percent change from MELD-Na to MELD-Na_90 was an increase of 9% (IQR, -4% to 55%).

### 3.3. Baseline Variables as Prognostic Factors

110 deaths (60%) were observed during the follow-up period. Median actuarial survival was 17.1 months, and 12 and 24-month actuarial survival rates were 61% and 39%, respectively.

**Figure 1:**
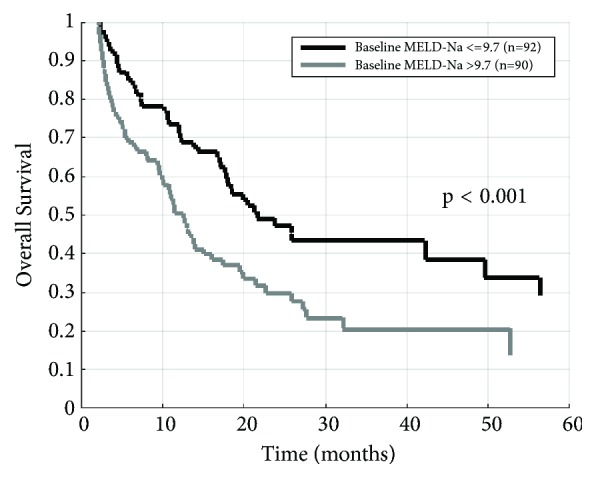
Kaplan-Meier overall survival curves after grouping patients by baseline MELD-Na. p value calculated using log-rank test.

Univariate and multivariable Cox modeling results identifying predictors of OS among baseline variables, including MELD-Na, are displayed in Table 2. In the multivariable model, clinical stage (HR=2.66 for stage III compared to stage I, 95% CI 1.64 to 4.32, p<0.001) and baseline MELD-Na (HR=1.05 per unit increase, 95% CI 1.01 to 1.09, p=0.012) were independently associated with increased mortality risk. Kaplan-Meier survival curves of patients by baseline MELD-Na are shown in Figure 1.

### 3.4. Change in MELD-Na as a Prognostic Factor

**Figure 2:**
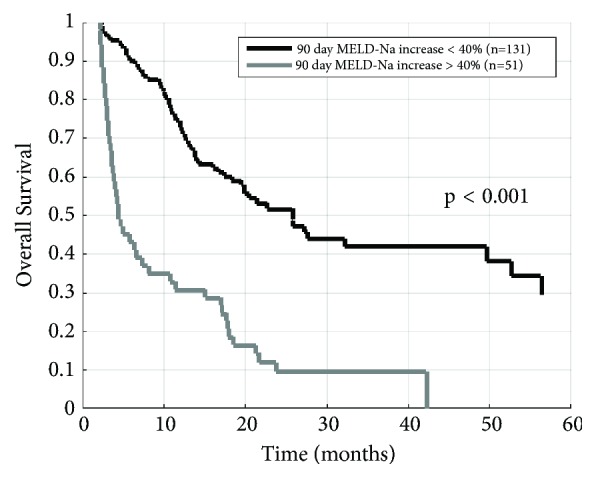
Kaplan-Meier overall survival curves after grouping patients using the optimal MELD-Na increase cutoff of 40%. p value calculated using log-rank test.

The optimal prognostic cut-point for 90-day increase in MELD-Na was found to be 40%. 51 patients (28%) demonstrated increases in MELD-Na exceeding 40%. Median survival among patients whose MELD-Na increased by more than 40% was 4.5 months, and median survival among the 131 other patients was 25.8 months (log-rank p<0.001, Figure 2).

A multivariable Cox proportional hazards model including baseline MELD-Na and the change in MELD-Na at 90 days demonstrated that both baseline MELD-Na (HR=1.07 per unit increase, 95% CI 1.03 to 1.11, p<0.001) and increase in MELD-Na exceeding 40% (HR=3.69, 95% CI 2.39 to 5.69, p<0.001) were independently associated with increased mortality risk. There was no evidence of interaction between these two measures as predictors of mortality (interaction term p=0.519).

**Figure 3:**
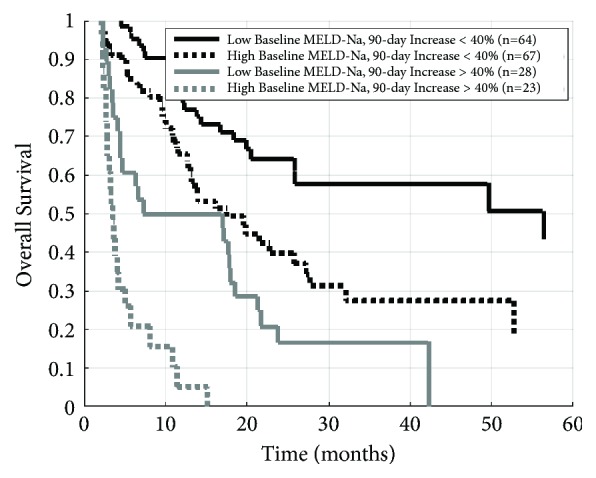
Kaplan-Meier overall survival curves after grouping patients by both baseline MELD-Na and MELD-Na increase. “Low” and “High” baseline MELD-Na groups defined as ≤ 9.7 or > 9.7, respectively.

Kaplan-Meier survival curves after dividing patients into four groups based on baseline MELD-Na and the change in MELD-Na are shown in Figure 3. Patients with baseline MELD-Na less than or equal to the median value of 9.7 whose MELD-Na increased by less than 40% at 90 days had the most favorable prognosis, with a median survival of 56.3 months. In contrast, median survival for patients with baseline MELD-Na exceeding 9.7 whose MELD-Na increased by more than 40% was only 3.6 months. As summarized in supplementary Table 1, these patients were more likely to die from both progression of their liver disease (p<0.001) and from cancer-related death (p=0.013) than patients whose MELD-Na increased by less than 40% at 90 days. In addition, these patients were more likely to receive either systemic therapy (18% versus 11%, p<0.001) or no therapy (29% versus 10%, p<0.001) in comparison to the group with more favorable prognosis as shown in Table 3.

## 4. Discussion

The present study evaluated the utility of MELD-Na and 90-day changes in MELD-Na scores as predictors of mortality in a predominantly HCV-infected, racially diverse population of patients with nonmetastatic HCC. We demonstrated that both measures are powerful prognostic factors in this patient population. Used in combination, these measures may further define HCC patient subgroups with strikingly different prognoses.

The MELD-Na score was first shown to predict mortality in a predominantly Asian and HBV-infected HCC cohort [11, 17]. This observation has not been replicated in a Western population, where HCC patients have a different demographic and etiologic profile. While both chronic HBV and HCV are risk factors for HCC, development of HCC in relation to stage of liver disease is somewhat different for both viruses, likely due to differences in carcinogenesis between HCV and HBV. We have demonstrated that MELD-Na at the time of diagnosis is likely a relevant prognostic factor in our patient population that may be incorporated into management decisions and used to counsel patients.

After verifying the utility of MELD-Na as a prognostic factor in our cohort, we additionally demonstrated that an increase in MELD-Na score after 90 days exceeding 40% was associated with increased mortality risk. The dramatic increase in MELD-Na velocity in combination with high baseline MELD-Na > 9.7 identified a group of nonmetastatic HCC patients that have extremely poor prognosis with median survival of fewer than 4 months in our cohort. These patients have higher rates of mortality from both cancer-related death and from worsening of underlying liver disease. An increase in MELD-Na score denotes worsening hepatic function, as increase in MELD or MELD-Na score is correlated with hepatocyte loss [5, 18]. Rapid increase in MELD-Na may be indicative of rapid disease progression impairing hepatocyte function and/or deterioration of liver function as a consequence of liver-directed therapy. This finding is evident when we evaluated the choice of initial treatment modality and cause of death within each group. The patients with rapid increase in MELD-Na are more likely to die from cancer-related death rather than from liver disease. Our findings are in agreement with other groups that had demonstrated patients with HCC can die from complications both due to their underlying liver disease as well as from cancer progression [19].

Previous reports have examined changes in MELD as predictors of overall survival in patients with cirrhosis. In one study, a rate of MELD increase exceeding 2.5 points per month was strongly associated with 6- and 12-month mortality [20]. For comparison, the 40% increase in MELD-Na that we found to be the optimal prognostic factor in the current study corresponded to an increase of approximately 1 point per month for most patients.

Recalculated MELD scores after treatment with selective internal radiation therapy have previously been shown to be prognostic for overall survival [21]. The hazard ratio associated with a unit increase in MELD in that study was similar to the hazard ratio associated with a unit increase in MELD-Na in the present analysis. Additional variables that merit further exploration include volumetric tumor burden and initial response to therapy [21].

The current study has several limitations that must be acknowledged. Some missing data (e.g., cirrhosis etiology) could not be found in this retrospective study. Available data did not allow us to determine if 90 days following diagnosis is the optimal time at which to assess for changes in MELD-Na. Imaging data to allow assessment of disease progression were at times unavailable, precluding the inclusion of this important variable in our survival models.

In conclusion, our findings provide evidence for the general applicability of the MELD-Na score as a prognostic indicator for HCC patients, regardless of etiology. We have identified the velocity of change over 90-days in MELD-Na score as a potentially important prognostic factor. Care must be taken in interpreting the data as many HCC patients on the liver transplant waiting list have low risk of dying, and overemphasizing the mortality risk may lead to further disparity in the liver allocation system [22, 23]. However, our findings have identified a potential subgroup of HCC patients that have a significantly worse prognosis when compared to the majority of the HCC population that is not captured based on the traditional MELD-Na score [7]. Validation of these findings using additional datasets is warranted.

## Figures and Tables

**Table 1:** Characteristics of the 182 subjects included in this study.

**Characteristic**	**N=182**
Age, mean (SD)	61.8 (11.1)

Gender, n (%)	
Male	133 (73%)
Female	49 (27%)

Stage, n (%)	
I	65 (36%)
II	51 (66%)
III	66 (36)

AFP (ng/mL)	
< 400	127 (70%)
> 400	55 (30%)

Race, n (%)	
White	107 (59%)
Black/African American	54 (30%)
Asian	5 (2%)
Other/Unknown	16 (9%)

Ethnicity, n (%)	
Not Hispanic/Latino	85 (47%)
Hispanic/Latino	97 (54%)

Etiology of cirrhosis, n (%)	
HCV	120 (66%)
HBV	13 (7%)
HBV + HCV	4 (2%)
Alcoholism	24 (13%)
Unknown	21 (12%)

MELD-Na, median (IQR)	9.7 (7.5 to 13.3)

MELD-Na_90, median (IQR)	11.8 (8.5 to 18.8)

**Table 2:** Cox proportional hazard models for overall survival. Multivariable models were built using a backwards stepwise procedure.

	**Univariate**		**Multivariable:** **Baseline MELD-Na Only**		**Multivariable:** **Baseline MELD-Na and MELD-Na change**	
**Characteristic**	**HR (95**%** CI)**	**p**	**HR (95**%** CI)**	**P**	**HR (95**%** CI)**	**P**
Age (per 10 years)	0.95 (0.82 to 1.11)	0.505	-	-	-	-

Gender						
Male	[reference]	-	-	-	-	-
Female	0.80 (0.51 to 1.24)	0.318				

Stage						
I	[reference]	-	[reference]	-	[reference]	-
II	1.17 (0.70 to 1.98)	0.535	1.35 (0.76 to 2.39)	0.310	1.52 (0.86 to 2.71)	0.153
III	2.68 (1.71 to 4.21)	<0.001	2.66 (1.64 to 4.32)	<0.001	2.24 (1.37 to 3.67)	0.001

AFP (ng/mL)						
< 400	[reference]	-	-	-	-	-
> 400	1.72 (1.16 to 2.56)	0.007				

Race						
White	[reference]	-				
Black/African American	1.15 (0.75 to 1.74)	0.525	-	-	-	-
Asian	0.26 (0.04 to 1.86)	0.179				
Other/Unknown	1.08 (0.52 to 2.26)	0.834				

Ethnicity						
Not Hispanic/Latino	[reference]	-	-	-	-	-
Hispanic/Latino	0.88 (0.61 to 1.28)	0.509				

Etiology of cirrhosis, n (%)						
HCV only	[reference]	-				
HBV only	1.60 (0.77 to 3.43)	0.209	-	-	-	-
Alcoholism	0.66 (0.35 to 1.25)	0.205				
Unknown/Multiple	0.77 (0.42 to 1.40)	0.385				

MELD-Na	1.06 (1.03 to 1.10)	<0.001	1.05 (1.01 to 1.09)	0.012	1.07 (1.03 to 1.11)	<0.001

90 day MELD-Na increase						
< 40%	[reference]	-	-	-	3.69 (2.39 to 5.69)	<0.001
> 40%	3.44 (2.29 to 5.17)	<0.001				

**Table 3:** Initial treatment modality grouped by MELD-Na increases cutoff of 40%. One patient had missing information regarding initial treatment modality. TACE = transcatheter arterial chemoembolization, RFA = radiofrequency ablation, Y-90 = yttrium-90 radioembolization, and SBRT = stereotactic body radiation therapy. Total percent may not add up to 100% due to rounding.

**Initial Treatment Modality Within 60 Days of Diagnosis**	**MELD-Na Increase from Baseline**	
	**<40**%**, n=131**	**≥40**%**, n=51**
**TACE, n (**%**)**	61 (47)	18 (35)

**RFA, n (**%**)**	18 (14)	5 (10)

**Sorafenib, n (**%**)**	14 (11)	9 (18)

**Resection, n (**%**)**	13 (10)	1 (2)

**Y-90, n (**%**)**	10 (8)	2 (4)

**SBRT, n (**%**)**	0 (0)	1 (2)

**Transplant, n (**%**)**	1 (1)	0 (0)

**None, n (**%**)**	13 (10)	15 (29)

## Data Availability

The patient data used to support the findings of this study are restricted by the Albert Einstein College of Medicine Institutional Review Board in order to protect patient privacy. Data are available for researchers who meet the criteria for access to confidential data.

## Abbreviations

HCC: Hepatocellular carcinoma
MELD: Model for end-stage liver disease
MELD: Na-Model for end-stage liver disease and sodium
HBV: Hepatitis B
HCV: Hepatitis C
INR: International normalized ratio
MMC: Montefiore Medical Center
PCR: Polymerase chain reaction
AFP: Alpha fetoprotein
TACE: Transcatheter arterial chemoembolization
RFA: Radiofrequency ablation
IQR: Interquartile range.

## Supplementary Materials

Supplementary Materials Table 1. Cause of death grouped by MELD-Na increase cutoff of 40%.
