# Supplementary material for: Model for End-Stage Liver Disease and Sodium Velocity Predicts Overall Survival in Nonmetastatic Hepatocellular Carcinoma Patients
Source: Can J Gastroenterol Hepatol. 2018 Nov 7;2018:5681979. doi: 10.1155/2018/5681979 (PMC6247644; doi:10.1155/2018/5681979)
Supplement: Supplementary Materials — Table 1. Cause of death grouped by MELD-Na increase cutoff of 40%. [file 5681979.f1.pdf]

Cause of Death	MELD-Na Increase from Baseline	
	**<40%, n=131**	**≥40%, n=51**
**Progression of Liver Disease, n (%)**	15 (11)	13 (25)
**Cancer Related Deaths, n (%)**	43 (33)	27 (53)

Supplementary Table 1. Cause of death grouped by MELD-Na increase cutoff of 40%.
